# Significance of cyclin D1 overexpression in progression and radio-resistance of pediatric ependymomas

**DOI:** 10.18632/oncotarget.23509

**Published:** 2017-12-20

**Authors:** Muh-Lii Liang, Tsung-Han Hsieh, Yun-Ru Liu, Yi-Wei Chen, Yi-Yen Lee, Feng-Chi Chang, Shih-Chieh Lin, Ming-Chao Huang, Ho Donald Ming-Tak, Tai-Tong Wong, Yun Yen, Muh-Hwa Yang

**Affiliations:** ^1^ Division of Pediatric Neurosurgery, Neurological Institute, Taipei Veterans General Hospital, Taipei, Taiwan; ^2^ Institutes of Clinical Medicine, National Yang-Ming University, Taipei, Taiwan; ^3^ Comprehensive Cancer Center of Taipei Medical University, Taipei Medical University, Taipei, Taiwan; ^4^ Joint Biobank, Office of Human Research, Taipei Medical University, Taipei, Taiwan; ^5^ Department of Oncology, Taipei Veterans General Hospital, Taipei, Taiwan; ^6^ Department of Radiology, Taipei Veterans General Hospital, Taipei, Taiwan; ^7^ Department of Pathology and Laboratory Medicine, Taipei Veterans General Hospital, Taipei, Taiwan; ^8^ Department of Neurosurgery, Taipei Medical University Hospital, Taipei Medical University, Taipei, Taiwan; ^9^ Neuroscience Research Center, Taipei Medical University Hospital, Taipei, Taiwan; ^10^ Institutes of Clinical Medicine, Taipei Medical University, Taipei, Taiwan; ^11^ PhD Program for Cancer Biology and Drug Discovery, College of Medical Science and Technology, Taipei Medical University, Taipei, Taiwan; ^12^ Research Center of Cancer Translational Medicine, Taipei Medical University, Taipei, Taiwan; ^13^ Cancer Research Center & Genome Research Center, National Yang-Ming University, Taipei, Taiwan; ^14^ Immunity and Inflammation Research Center, National Yang-Ming University, Taipei, Taiwan; ^15^ Division of Hematology-Oncology, Department of Medicine, Taipei Veterans General Hospital, Taipei, Taiwan; ^16^ Genomic Research Center, Academia Sinica, Taipei, Taiwan

**Keywords:** ependymoma, pediatric, radio-resistance, CCND1

## Abstract

Due to the limited efficacy of chemotherapy, the applications of adjuvant irradiation play an important role for ependymoma treatment. However, in the young ages, the resistance of residual and recurrent tumor, and long-term intellectual sequelae remain the major obstacles of radiotherapy. Understanding the mechanism of therapeutic failure caused by radio-resistance is, therefore, crucial in ependymoma treatment. Here we retrospectively analyze clinic-pathological factors in 82 cases of ependymoma less than 20 years old and identify radio-resistant genes through gene expression microarray followed by qRT-PCR validation and immunohistochemistry staining. Thirty-one out of 82 (37.8%) patients are under 3-year-old. The 10 years PFS and OS are 38% and 60%. Gross-total resection is the single significant prognostic factor for longer 10 years PFS and OS in the multivariant analysis (*p<0.05*). According to the microarray analysis, CCND1 is up-regulated in supratentorial and infratentorial ependymomas and is associated with DNA repair. We demonstrated that 24 primary and 16 recurrent ependymomas were up-regulated, and 5 out of 7 paired samples exhibited higher *CCND1* expression in recurrent tumors. We also found RAD51, another DNA repair gene, was up-regulated in supratentorial and infratentorial ependymomas. Knocking down CCND1 reduced cell proliferation and repressed several genes associated with S-phase and DNA repair. Homologous recombination activities of DNA repair were significantly decreased in CCND1-deficient cells while the level of γH2AX was increased after irradiation. In summary, these observations suggest a robust role of CCND1 in regulating cell proliferation and radio-resistance in ependymomas, providing a potential therapeutic target for pediatric ependymomas.

## INTRODUCTION

Intracranial ependymoma is one of the most malignant brain tumors in children, which performs only 45-75% 10 years survival rate [1–3]. Although maximal safe resection combined adjuvant chemo-radiation therapy provides effective treatment, many patients sustain the recurrence of the residual tumors. The challenges to cure ependymomas include young age, dorsal brainstem complication, cerebellopontine angle and lateral medullary cranial nerves expansion, important vessels encasement, craniospinal seeding, and chemo- & radio-resistance. Therefore, a robust therapeutic strategy for ependymoma should aim for gross-total resection and conformal postoperative irradiation at doses at least exceed 45Gy [2, 4]. The advantage of pre-irradiation chemotherapy in patients with the residual tumor has been demonstrated in COG study, which is restricted to the patients with >90% resection or <1.5cm^2^ residual [5]. Despite the tremendous efforts, more than 20% cases remain recurrent due to the insensitivity of radiation and chemotherapeutic agents [6].

The clinical outcome with high-dose chemotherapy treatment of ependymomas has not been proven yet. Several studies have focused on understanding the mechanisms that lead to chemotherapy insensitivity in ependymoma. Specifically, ATP-binding cassette sub-family B member 1 (ABCB1, also known as multidrug resistant protein 1(MDR1) or P-glycoprotein 1) upregulation has been characterized as a potential mechanism [7]. Furthermore, O6-Methylguanine-DNA-methyltransferase (MGMT) upregulation and reduced promoter methylation have also been reported in recurrent ependymomas [8]. Compared with astrocytic tumors, primary ependymomas also showed lower methylation at MGMT promoter [9]. However, little has known about the radio-resistance in ependymomas. Bobola et al. suggested that the level of apurinic/apyrimidinic endonuclease (Ap endo) activity after radiotherapy negatively correlated with the survival rate of progression-free and overall samples [10]. The underlying mechanism of radio-resistance in ependymomas remains elusive.

Homologous recombination (HR) is one of the mechanisms involved in repairing radiation-induced double strand DNA breakage (DSB). When DSB occurs, protein RPA coats on the single-stranded DNA, recruiting RAD51 recombinase (RAD51) and breast cancer 2 (BRCA2) to perform HR [11]. Previous studies found that Cyclin D1 (CCND1) was also recruited to the I-SceI-induced double-stranded DNA break sites [12]. CCND1 associates with RAD51 and BRCA2 after the generation of DSB. The recruitment of RAD51 at DSB sites disappeared after knocking down CCND1. In brief, BRCA2 recruits CCND1 to the DNA damage sites, which then engages with RAD51 through a direct CCND1–RAD51 interaction. On the other hand, Marampon et al. also found that, in prostate cancer, CCND1 would bind to RAD51 after the radiation treatment [13]. CCND1 also interacts with chromatin-modifying enzymes and several transcriptional factors to regulate proliferation and differentiation, such as Cyclin D-cyclin-dependent kinase 4 (CDK4) and CDK6 [14].

By analyzing high throughput gene expression microarray followed by qRT-PCR validation of ependymoma tissues, we proved CCND1 and RAD51 upregulation in both primary and recurrent ependymomas. CCND1 expression showed upregulation after 7 days irradiation in ependymoma cells. Knocking down CCND1 in ependymoma cells suppressed the activities of irradiation-induced homologous recombination and cell proliferation. In summary, the study highlights the limitations of current adjuvant therapies and the important role of CCND1 in ependymoma. In addition, we also demonstrated a potential radio-resistance mechanism of which enhances the DNA repair in pediatric ependymomas cells.

## RESULTS

### Clinical characterization

82 ependymoma patients were enrolled and the male to female ratio is 1.16 (48 to 38). Mean age is 6.2 years old. Seven cases were initially diagnosed before one year of age, and 31 cases were less than 3 years old. Two cases were initially diagnosed as Grade II then changed to Grade III at second operation. Gross total resection was achieved in 47 patients (57%). The post-operative adjuvant treatment was performed, including radiation alone on 34 patients (41%), chemo-radiation on 32 patients (39%), and non-adjuvant treatment or chemotherapy only on 16 patients (20%). The mean follow-up period was 87 months (ranging from 2 to 301 months) (Table 1).

**Table 1:** Clinical Characteristics of 82 children treated for ependymoma

Factors	No. of patients (%)	Prognostic value (*P* value)
**Sex**		0.7901
male	44 (53)	
female	38 (47)	
**Age** (yrs)		0.8447
<3	31 (37)	
>=3	51 (63)	
**Location**		0.0001^*^
supratentorium	24 (29)	
posterior fossa	53 (65)	
spinal	5 (6)	
**WHO grade**		0.6226
I	2 (2)	
II	26 (32)	
III	54 (66)	
**Hazard location**		0.0001^*^
yes	38 (48)	
no	42 (52)	
**Extent of Surgery**		0.0003^*^
gross total	35 (43)	
non-total	47 (57)	
**Adjuvant treatment**		
non- or chemotherapy alone	16 (20)	
radiation alone	34 (41)	0.0006^*^
radiation and chemotherapy	32 (39)	0.1582
**MIB-1 index§**		0.0138^*^
>=50	12 (32)	
<50	25 (68)	
**Recurrence ₸**		NA
focal	16 (38)	
leptomeningeal seeding	27 (62)	

^*^: statistically significance on univariant analysis for 10 years overall survival

§: total 37 cases reviewed,

₸: total 43 cases reviewed during follow up period

NA: not available

### Clinical factors for survival and progression

**Figure 1:**
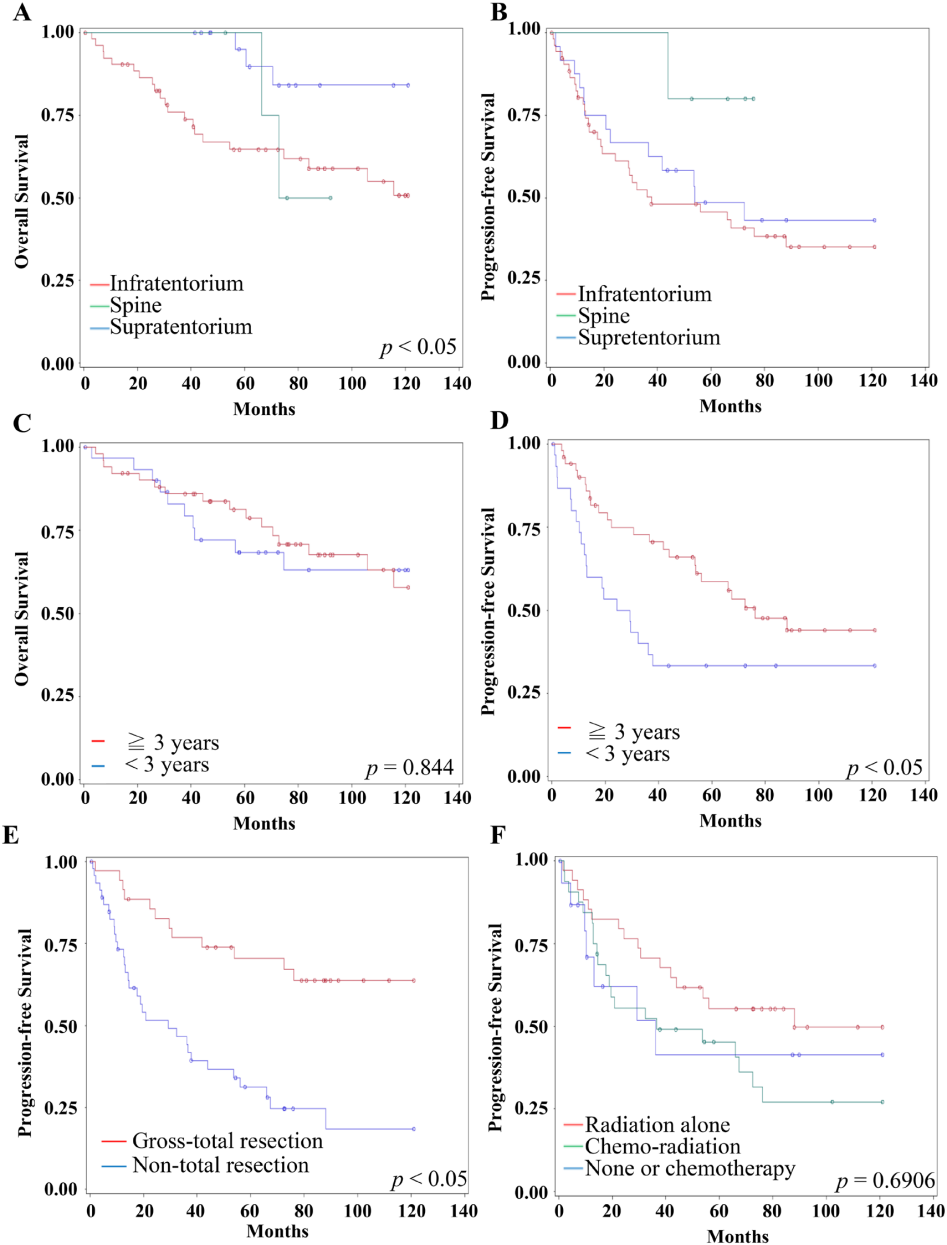
Clinical factors for Survival and Progression **(A-B)** The 10 years overall survival (A) and progression-free-survival (B) of different locations were distinct. Red line represents infratentorium. Green line represents spine. Blue line represents supratentorium. **(C)** The 10 years overall survival for more than or equal to 3 vs. less than 3-year-old patients were no significant (*P=*0.844). Red line represents equal or more than 3-year-old. Blue line represents less than 3-year-old. **(D)** The age less than 3-year-old was significantly related to worse 10 years progression-free survival (*P=*0.040). Red line represents equal or more than 3-year-old. Blue line represents less than 3-year-old. **(E)** The gross total resection was significantly related to a longer 10 years progression-free survival (*P=*0.003). Red line represents gross-total resection. Blue line represents non-total resection. **(F)** There is no significant difference of 10 years progression-free survival among adjuvant treatment by using Kaplan Meier method (*p*=0.63). Red line represents adjuvant radiation alone (N=41). Green line represents adjuvant chemo-radiation (N=39). Blue line represents none or chemotherapy alone (N=20).

The 10 years PFS and OS were 38% and 60 %, respectively. The survival rates differ with distinct anatomic locations. For example, supratentorial ependymoma reveals 83% 10 years OS, while posterior fossa and spine location show 59% and 100%, respectively (*P<*0.05)(Figure 1A). The 10 years PFS for three locations were 43%, 35% and 80% (Figure 1B). The 10-years OS and PFS for more than or equal to 3 vs. less than 3-year-old patients were 63% & 44% vs. 57% & 33%, respectively (*P=*0.844 for OS, *P=*0.040 for PFS)(Figure 1C and 1D).

On univariant analysis, hazard location (*P<*0.001), non-radiation therapy (*P=*0.006) significantly associated with a shorter 10 years OS. The gross total resection (*P=*0.003), MIB-1 indices less than 50 *(P=*0.0138) significantly associated with a longer 10 years OS (Supplementary Figure 1). Four factors were significantly associated with shorter 10 years PFS: less than 3-year-old (*P=*0.0408), WHO grade III (*P=*0.0136), hazard location (*P<*0.0001), and non-total resection (*P=*0.0003)(Figure 1E). Refining the significant clinical variables with a Cox proportional hazards model suggested that gross total resection was the single significant prognostic factor for longer 10 years PFS and OS in multivariant analysis (RR=0.35, 95% CI 0.138-0.883; *P=*0.0263 and RR=0.111, 95% CI 0.019-0.654; *P=*0.0152) (Table 2). The additional irradiation or chemo-radiation had significant survival benefit; however, those adjuvant treatments did not achieve statistic difference on 10 year PFS (*P*=0.6906) (Figure 1F). These data reflected the resistance of the residual ependymomas to the adjuvant chemo-radiation therapy.

**Table 2:** Multivariant analysis for relative risks of shorter 10 years progression free survival and overall survival estimated with a Cox proportional hazard model

Clinical factors	10 years progression free survival			10 years overall survival		
	P value	RR	95% CI	P value	RR	95% CI
Sex (male)	0.0190^*^	0.464	0.245-0.882	0.0199^*^	0.372	0.161-0.855
Age < 3Y	0.3175	1.411	0.718-2.773	0.1041	0.456	0.177-1.176
WHO grade (II, III)	0.0714	1.967	0.943-4.105	0.9885	1.006	0.425-2.382
Hazard location	0.3077	1.592	0.652-3.890	0.0901	2.869	0.848-9.708
Gross total resection	0.0263^*^	0.350	0.138-0.883	0.0152^*^	0.111	0.019-0.654
Adjuvant treatment						
Irradiation	0.6906	0.843	0.363-1.958	0.0028^*^	0.135	0.036-0.502
Chemo-radiation	NA	NA	NA	0.0081^*^	0.254	0.092-0.700

^*^: statistically significance (*p*<0.05)

RR: relative risk, CI 95%: confidential interval, WHO grade II: classical, WHO III grade III: anaplastic, NA: not calculated due to no significance on univariant analysis.

### Differentially expressed genes between normal and ependymomas

**Figure 2:**
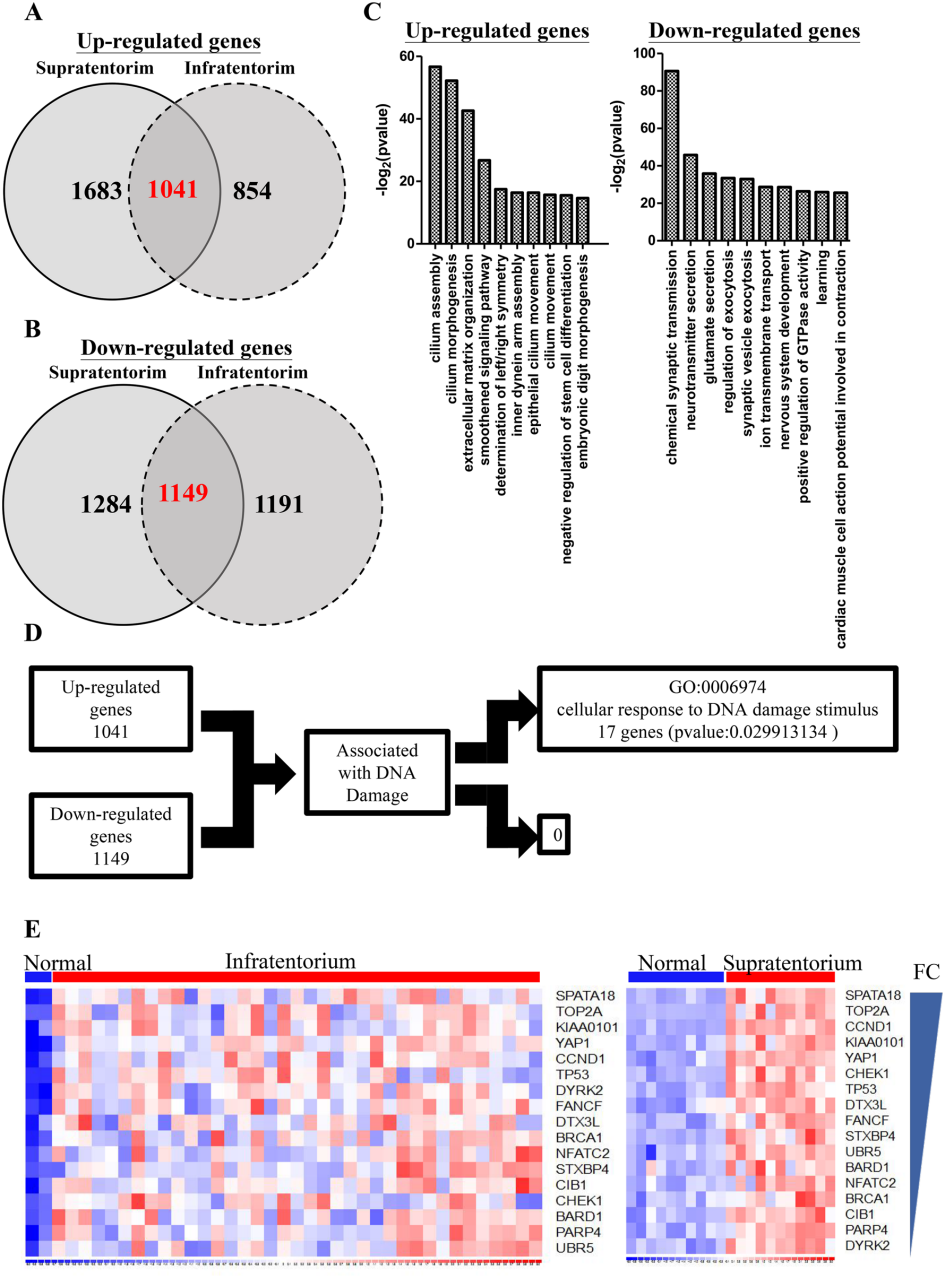
Differentiated genes in infratentorium and supratentorium ependymomas **(A-B)** Venn diagram show the up-regulated (A) (q <0.05, Fold change ≧ 2) or down-regulated (B) (q <0.05, Fold change ≦ 2) genes in supratentorium (left) and infratentorium (right) compared with normal tissues. The numbers of the intersections indicate the overlapped genes across all groups. **(C)** Up-regulated (left panel) and down-regulated (right panel) genes in supratentorium and infratentorium were subjected to Gene Ontology (GO) database searches. **(D)** Schematic representation for identifying DNA damage associated targets. The putative targets were obtained from up-regulated or down-regulated genes which are associated with DNA damage response. **(E)** Heatmap showed that up-regulated genes were associated with DNA damage response and sorted by fold change. (left: infratentorium, right: supratentorium).

43 ependymoma samples including 21 supratentorium and 22 infratentorium location were enrolled in the study. Fourteen primary and seven recurrent ependymoma samples located on supratentorium, and twelve primary and ten recurrent ependymoma samples located on infratentorium. Since the therapeutic resistance of residual tumors is the major cause of recurrent ependymomas, exploring genes associated with the recurrent tumors is, therefore, important for ependymoma treatment. We combined our microarray datasets (2 supratentorium and 2 infratentorium) and GSE66354 from GEO database to analyze the differential expression profiles. Since previous studies suggested that the different locations of tumors (supratentorial and infratentorial) would show distinct expression profiles [15], we analyzed these samples derived from different anatomic location separately. 2724 and 2433 genes showed increased and decreased in supratentorial ependymomas, respectively (q<0.05, Fold change≧2)(Figure 2A & 2B). 1895 and 2340 genes showed increased and decreased in infratentorial ependymomas (q<0.05, Fold change≧2)(Figure 2A & 2B). By intersecting the genes up-regulated or down-regulated in both parts of ependymoma, we found 1041 and 1149 genes simultaneously up-regulated and down-regulated in both datasets (Figure 2A & 2B). Gene Ontology analysis showed up-regulating genes participating in cilium assembly, cilium morphogenesis, and extracellular matrix organization (Figure 2C, left). By contrast, down-regulating genes got involved in chemical synaptic transmission, neurotransmitter secretion, and glutamate secretion (Figure 2C, right). Radiotherapy commonly applied in killing tumors and the damages of tumor cells DNA leads to cellular death. To identify the genes associated with DNA damages, GO analysis showed 17 up-regulating and none of the down-regulating genes in response to DNA damage stimulus (Figure 2D & 2E).

### Overexpression of cyclin D1 in primary and recurrence ependymomas

**Figure 3:**
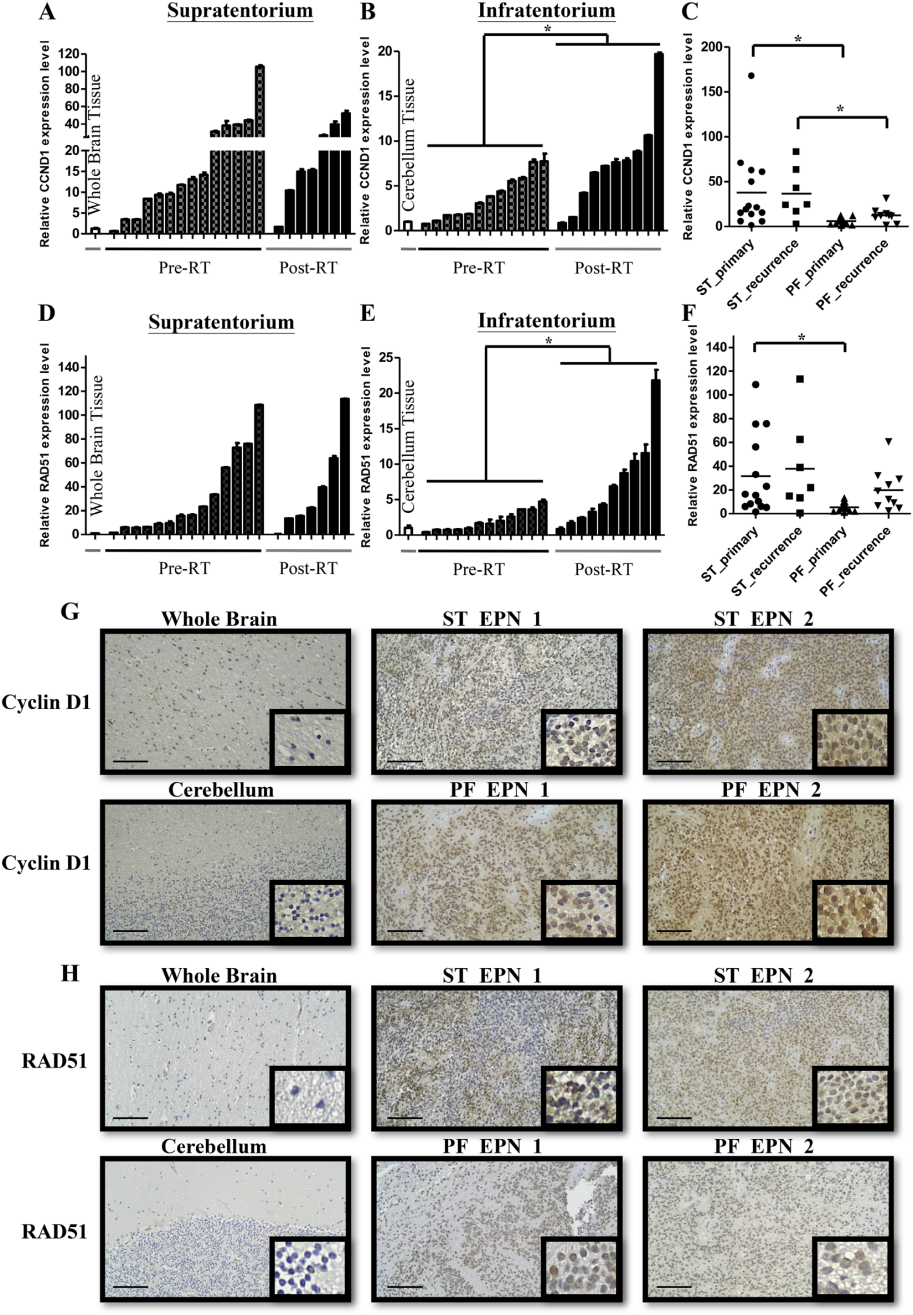
CCND1 and RAD51 are overexpressed in primary and recurrent ependymomas **(A-B)** CCND1 expression validated in primary (Pre-RT) and recurrent (Post-RT) supratentorium (A) and infratentorium (B) compared with normal whole brain tissue and cerebellum, respectively. qRT-PCR results are presented as mean±SD for duplicate samples. **(C)** CCND1 expression was more in primary and recurrent supratentorium than infratentorium. ^*^p<0.05 by t-test. **(D-E)** RAD51 expression validated in primary and recurrent supratentorium (D) and infratentorium (E) compared with normal whole brain tissue and cerebellum, respectively. qRT-PCR results are presented as mean±SD for duplicate samples. **(F)** RAD51 expression was more in primary supratentorium than infratentorium. ^*^p<0.05 by t-test. **(G-H)** IHC analyses confirmed protein levels of CCND1 (G) and RAD51 (H) in supratentorium and infratentorium. ST: supratentorium, PF: infratentorium. Scale bar: 100 μm.

**Figure 4:**
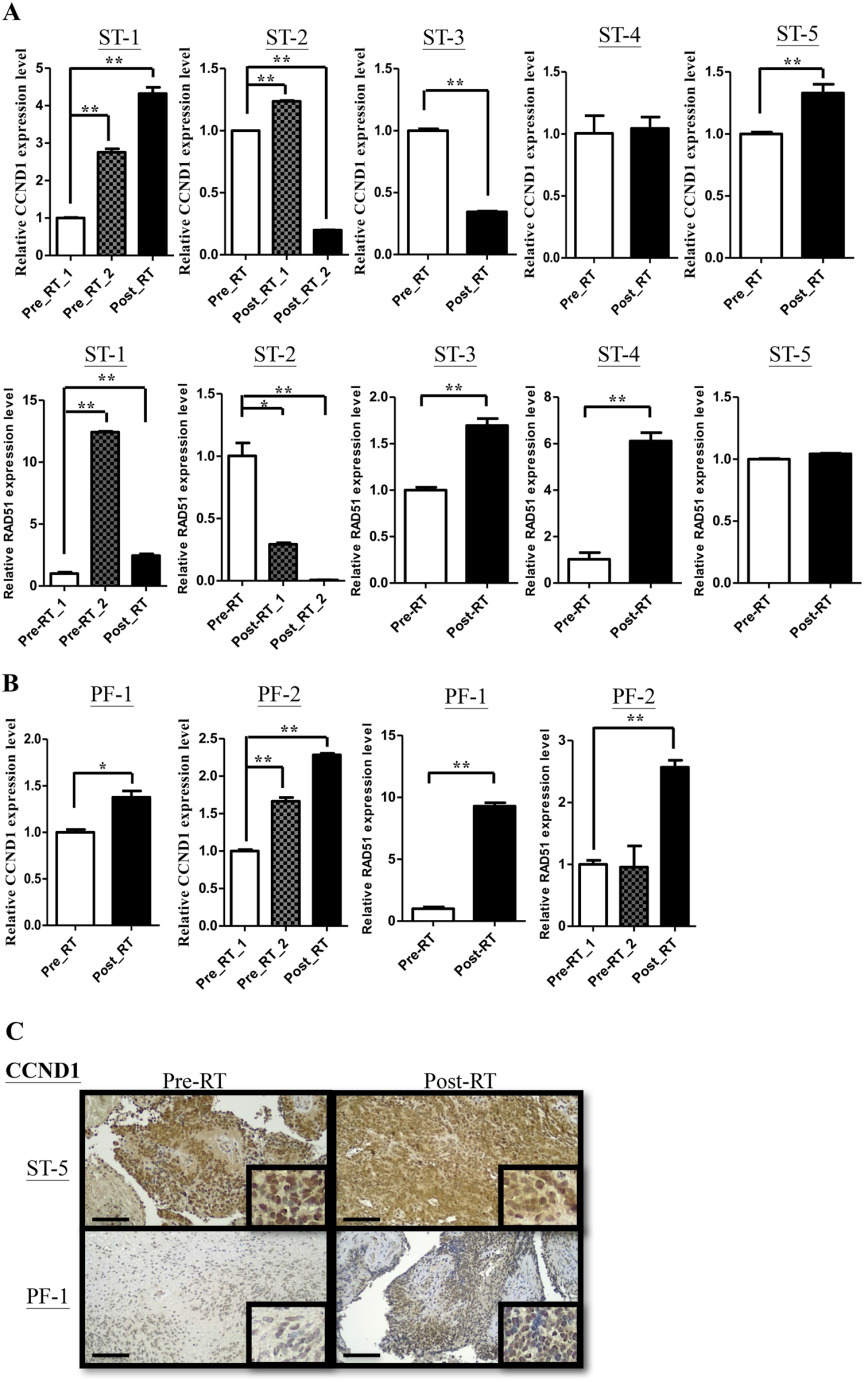
CCND1 and RAD51 are overexpressed in paired recurrent ependymomas **(A-B)** qRT-PCR analyses confirmed CCND1 (ST: upper panel, PF: left panel) and RAD51 (ST: lower panel, PF: right panel) expression in paired samples (pre-RT v.s. post-RT) from supratentorium (A) and infratentorium (B). ST: supratentorium, PF: infratentorium. **(C)** IHC analyses confirmed protein levels of CCND1 in paired samples (pre-RT vs. post-RT). Scale bar: 100 μm.

In supratentorial and infratentorial tumors, CCND1, one of the top 5 genes, expressed higher average level than others (SPATA18, TOP2A, KIAA0101 and YAP1). CCND1 has been found contributing to DNA repair in previous studies [12, 13, 16]. To confirm whether CCND1 were associated with DNA repair in ependymomas, we checked the expression level of CCND1 using qRT-PCR and IHC staining firstly. Compared with whole brain tissue, among 14 primary and 7 recurrent supratentorial tumors, 13 (92%) primary and 7 (100%) recurrent tumors showed upregulation (Figure 3A). On the other hand, compared with normal cerebellum, among 12 primary and 10 recurrent infratentorial tumors, 11 (92%) primary and 9 (90%) recurrent tumors were up-regulated (Figure 3B). Furthermore, primary and recurrent tumors in supratentorium exhibited higher CCND1 level than primary and recurrent tumors in infratentorium, respectively (Figure 3C). Previous studies demonstrated that CCND1 bound to BRCA2 and RAD51 after radiation exposure to perform homologous recombination (HR). We then measured the relative expression level of RAD51 in each sample. In 14 primary and 7 recurrent supratentorial tumors, 14 (100%) primary and 6 (86%) recurrent tumors were up-regulated when compared with whole brain normal tissue (Figure 3D); out of 12 primary and 10 recurrent infratentorial tumors, 7 (58%) primary and 9 (90%) recurrent tumors were up-regulated when compared with normal cerebellum (Figure 3E). Primary tumors in supratentorium have higher RAD51 expression than infratentorium (Figure 3F). The protein level of CCND1 and RAD51 also showed intermediate or strong intensity comparing to normal tissue on IHC staining (Figure 3G and 3H). To elucidate the prognostic effect of CCND1, the cutoff value was defined as the medium of the total relative expression of CCND1 (N=45). However, the expression of CCND1 did not achieve the significant statistic difference on 10 years overall survival (*P*=0.2902; Supplementary Figure 2). Next, we want to know whether CCND1 expression is up-regulation in paired recurrent tumors. We quantified CCND1 mRNA and protein level in seven paired samples (5 supratentorial and 2 infratentorial tumors, including pre-& post-RT ependymomas tissue due to relapse of the same case) and found higher CCND1 level in recurrent tumors from 3 supratentorial and 2 infratentorial tumors (Figure 4A, 4B and 4C). One case (ST-3) had decreased expression on recurrent tumor and one case (ST-4) had no significant difference (Figure 4A and 4B). In terms of RAD51, we also found higher RAD51 level in recurrent tumors from 2 paired supratentorial and 2 paired infratentorial tumors (Figure 4A and 4B).

### CCND1 is associated with cell proliferation and DNA repair in ependymoma

**Figure 5:**
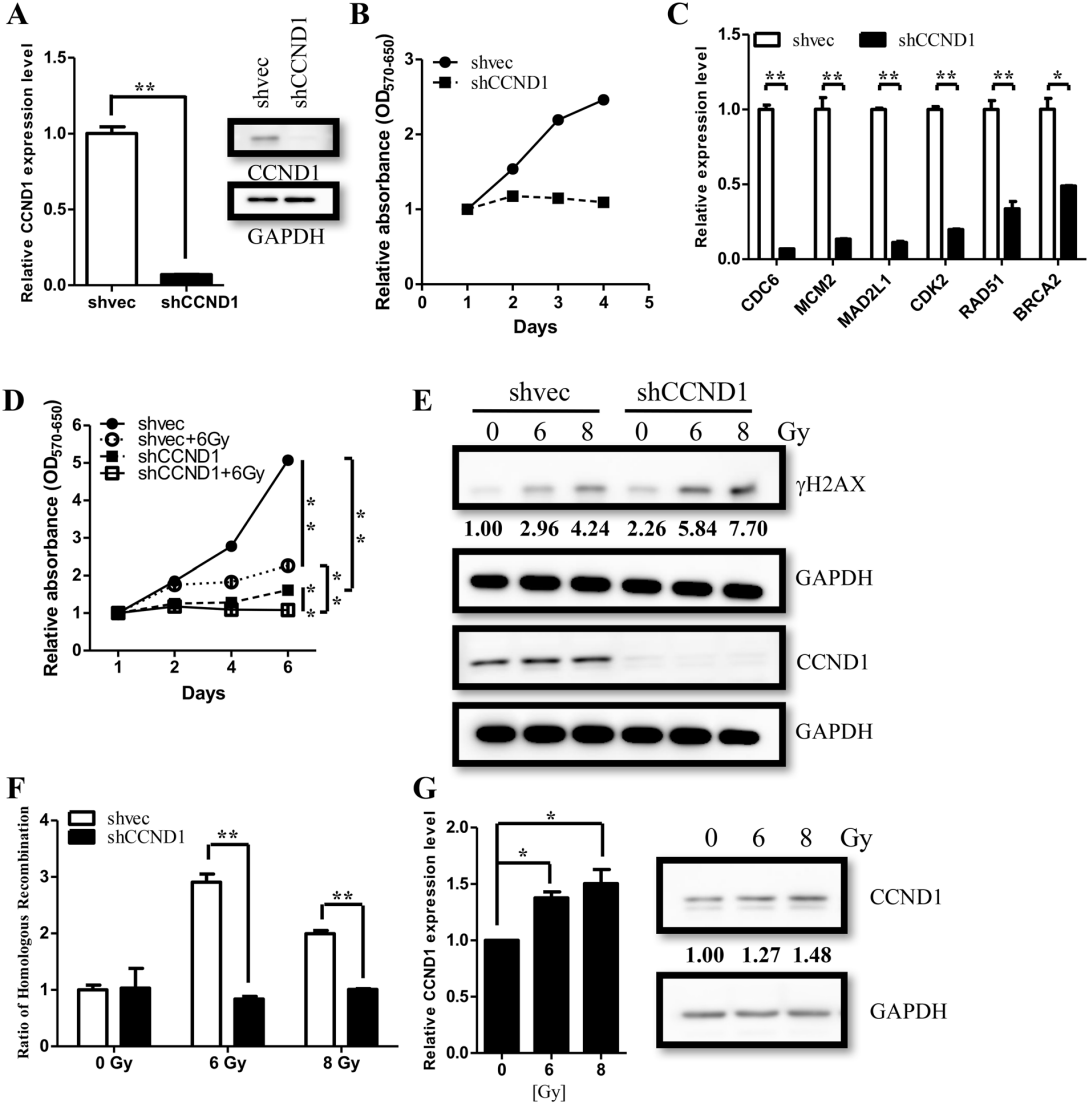
CCND1 regulate cell proliferation and DNA repair in ependymomas **(A)** Knocking downCCND1 (shCCND1) expression in ependymoma cells, which was confirmed through qRT-PCR and immunoblotting. **(B)** Knocking down CCND1 expression decreasedcell proliferation rate in ependymoma cells asmeasured by MTT assay. **(C)** Significant suppression of downstream genes was validated by qRT-PCR. qRT-PCR results are presented as mean±SD for duplicate samples.^**^p<0.01 by t-test. **(D)** Cell proliferation rate were measured after radiation treatment (6Gy). **(E)** Strong γH2AX expression level indicates DNA damage after radiation treatment, and be enhanced in shCCND1. **(F)** Knocking down CCND1 decreased DNA repair in ependymoma cells after radiation treatment (6 Gy and 8Gy) as measured by homologous recombination assay. **(G)** CCND1 expression were measured after radiation treatment (6 Gy and 8 Gy). qRT-PCR results are presented as mean±SD for duplicate samples.^**^p<0.05 by t-test.

To elucidate the role of CCND1, we knocked down CCND1 in two primary ependymoma cells and determined the proliferation by MTT assay (Figure 5A, Supplementary Figure 3A). Knocking down CCND1 resulted in a lower proliferation rate as compared to the vector control (Figure 5B, Supplementary Figure 3B). CCND1 were known to be associated with CDK4/6 to phosphorylate pRB, which allows E2F protein to regulate S phase genes expression [16]. We then measured S phase genes including CDC6, MCM2, MAD2L1, CDK2, and other DNA repair genes such as BRCA2 and RAD51. We found that knocking down CCND1 decreased the expression of these genes (Figure 5C, Supplementary Figure 3C). These data demonstrated that the CCND1 deficiency and the sequential S phase genes down-regulation lead to lower proliferation. To identify whether CCND1 causes radio-resistance in ependymomas, we knocked down CCND1 expression and quantified the corresponding cell viability after treatment with radiation. Treating CCND1-reducing cells (shCCND1) with radiation (6Gy) significantly reduced their proliferation rate as compared to the untreated and the irradiated control cells (shvec) (Figure 5D). We also found that the irradiated control cells remained proliferative regardless of a slow-down activity (Figure 5D). Furthermore, in CCND1-reducing cells, irradiation significantly induced DNA damage and revealed higher γ-H2AX levels, a biomarker for DNA double-strand breaks (Figure 5E). Noticeably, knocking down CCND1 lead to the downregulation of BRCA2 and RAD51 (Figure 5C, Supplementary Figure 3C), which was concordant with the previous discovery of the association between CCND1, BRCA2, and RAD51 after the radiation treatment. To confirm the effect on DNA repair after knocking down CCND1, we further detected homologous recombination efficiency after radiation exposure (see method). We co-transfected the provided plasmids (dl-1 and dl-2 plasmids) into CCND1-reducing and control cells. Compared with the control cells, the intensity of homologous recombination products were significantly lower after the radiation treatment (Figure 5F). In addition, we also found CCND1 upregulation after the 7 days high-dose irradiation (Figure 5G). These data indicated that silencing CCND1 induced DNA damage and abolished the repairing mechanisms after the radiation treatment.

### CDK4/6 inhibitor, palbociclib, reduced cell proliferation in ependymoma

**Figure 6:**
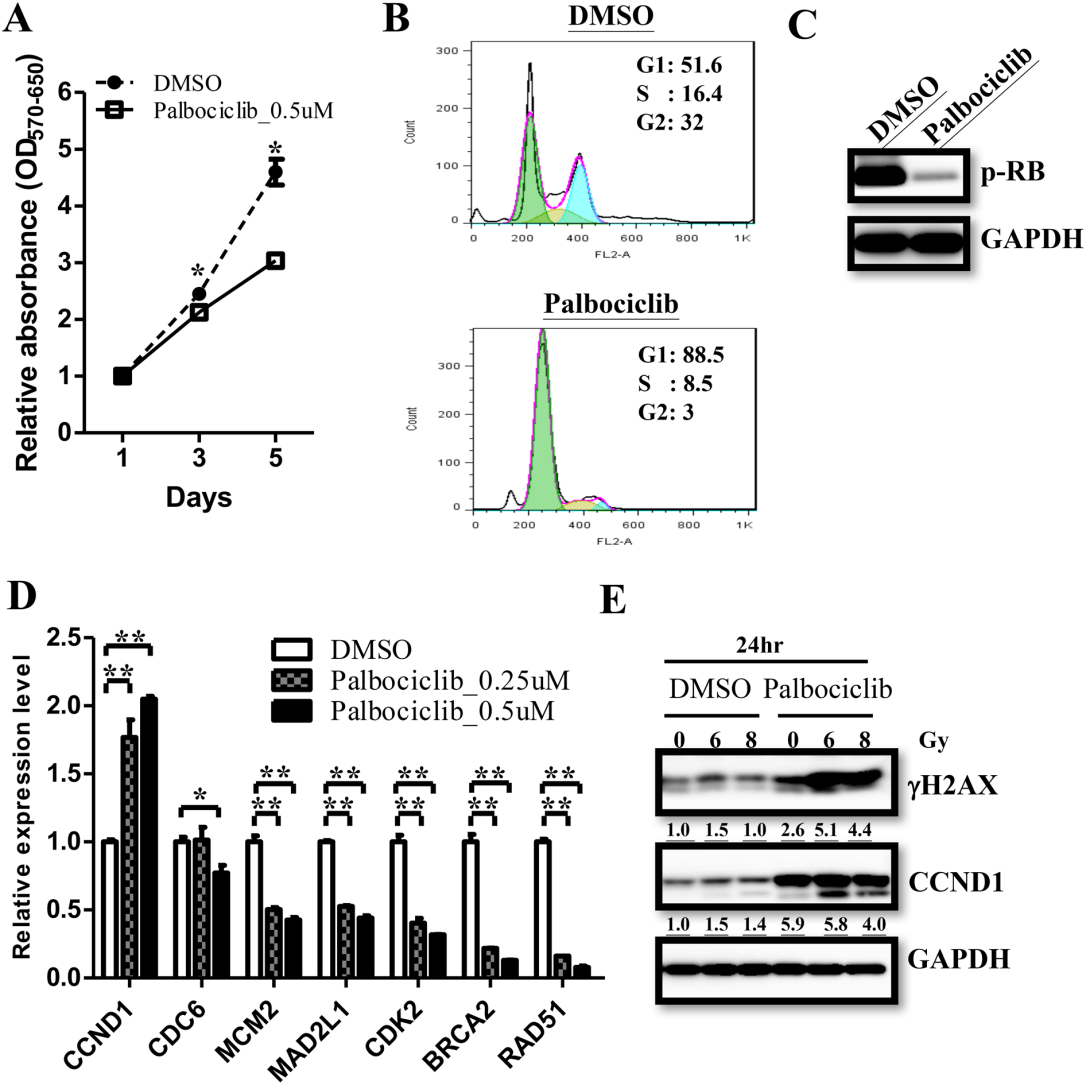
Palbociclib treatment regulate cell proliferation in ependymomas **(A)** Palbociclib treatment decreasedcell proliferation rate in ependymoma cells asmeasured by MTT assay. **(B)** Flow cytometry showed accumulated cells in the G1 phase after palbociclib treatment. **(C)** Palbociclib treatment decreased RB phosphorylation in ependymomas. **(D)** Significant suppression of downstream genes was validated by qRT-PCR. qRT-PCR results are presented as mean±SD for duplicate samples.^**^p<0.01 by t-test. **(E)** Prolonged γH2AX expression level indicates DNA damage after radiation treatment, and be enhanced in palbociclib treatment.

FDA has approved palbociclib (IBRANCE Capsules, Pfizer, Inc.) since Feb. 2016 by selectively targeting CDK4/6 in combination with fulvestrant to treat women with hormone receptor (HR)-positive, human epidermal growth factor receptor 2 (HER2)-negative advanced or metastatic breast cancer with disease progression. The advancement robustly supports the strategy that targeting the cyclin/CDK pathway for a more effective ependymoma treatment. The treatment efficiency of Palbociclib has been evaluated on AT/RTs and GBMs in previous studies [17, 18]. They described that the combination of palbociclib with radiation could sustain γH2AX expression and prevent tumor re-growth after treatment, suggesting palbociclib as a promosing treatment for ependymomas. To elucidate the treatment efficacy of palbociclib, we treated primary ependymoma cells with 0.5 μM palbociclib and determined the proliferation by MTT assay. Palbociclib treatment resulted in a lower proliferation rate as compared to the control (Figure 6A). Flow cytometry showed that palbociclib treatment accumulated cells in the G1 phase of the cell cycle and inhibited RB phosphorylation (Figure 6B & 6C). We then measured CDC6, MCM2, MAD2L1, CDK2, BRCA2 and RAD51 expression and found that decreased the expression of these genes after palbociclib treatment (Figure 6D). Furthermore, irradiation plus palbociclib treatment significantly induced higher γ-H2AX levels after 24 hours (Figure 6E). However, the intensity of homologous recombination products were no difference after the radiation treatment (data not shown). These data indicated that palbociclib treatment caused S phase genes down-regulation lead to lower proliferation and enhanced γ-H2AX levels after radiation treatment.

## DISCUSSIONS

This retrospective study comprises a large homogenous cohort of pediatric ependymomas. Throughout the period of three decades, they were treated according to the consensus of pediatric neuro-oncological team in Taipei Veterans General Hospital, ensuring treatment modalities with little changes and certain long-term outcome. In our cohort, univariant analysis identified non-hazard location, gross total resection, and adjuvant irradiation as being associated with favorable 10-year overall survival (Table 1). After analyzing clinical variables that had *P* value <0.05 on univariant analysis by using a Cox proportional hazards model, we suggested that only gross total resection was the significant factor for a longer 10-year PFS and OS (Table 2).

Despite the facts that many debates regarding the clinical factors influencing the treatment options in the literatures, the safe maximal resection with the aim of gross total resection is the prerequisite for highest overall and progression-free survival. Conformal high-dose (>54Gy) post-operative radiotherapy had been demonstrated to enhance the control of localized ependymoma, with high 5 years event-free survival to 74% in a prospective study [2]. However, the benefit of radiation has also been debated for their heterogeneous modalities that make comparison difficult [2, 4, 19]. The long-term sequelae of intelligent after irradiation are the major concerns for the young children especially for those less than 3 years. Our clinical results highlighted the limitation of adjuvant treatment that made high relapse rate of the resistant residual ependymomas tumors and the importance of developing new approach for radiation-related factors.

The histological feature had been reported as either a prognostic marker or a trend of worse outcome in the literature [2, 20]. In a retrospective review of 33 pediatric infratentorial ependymoma, the anaplastic histology was reported as the important factors affecting worse progression-free survival [21]. The WHO grade III with anaplastic histology in our study also reveals the trend of shorter progression-free survival in multivariant analysis (*P*=0.0714, RR 1.967, 95% CI 0.943-4.105). In the same review literature, Ki-67 indices were significant higher (13.1±9.4 vs. 1.2±2.4, *p*<0.001) in patients who experienced disease progression. MIB-1 indices (37 cases available) in our study, analyzed based on the literature, demonstrated a significant worse 5-year OS if the patients had MIB-1 indices level ≥50 than < 50 (*P*=0.0138)

CCND1, a putative oncogene on chromosome 11q13, is amplified and overexpressed in many neoplasms, such as squamous cell carcinoma in head and neck [22], ovarian cancer [23], breast cancer [24], and neuroblastomas [25]. Over-expression of CCND1 or amplification at the 11q13 region is also associated with poor prognosis and relapses in high-grade gliomas (normal brain tissues, low-grade gliomas, and high-grade gliomas were 4/18, 15/32, and 18/24), oligodendrogliomas, and ependymomas on supratentorial locations [26–28]. In the analysis of a large group of pediatric embryonal brain tumors including medulloblastoma and supratentorial neuroectodermal tumor (sPNET), CDK6 and CCND1 gene amplification are more commonly observed in sPNET (25%)[29]. When we referred to our other pediatric brain tumor datasets, including high-grade gliomas (HGGs), atypical teratoid/rhabdoid tumors (AT/RTs), and medulloblastoma (MBs), we found that CCND1 was also overexpressed in HGGs and AT/RTs (data not shown). HGGs and AT/RTs are highly malignant and recur more frequent than ependymoma and MBs. Whether CCND1 overexpression is associated with the malignancy and recurrence on HGGs and AT/RTs will require further investigation. In this study, we validated the higher level of CCND1 and RAD51 in ependymoma especially the tumors located on supratentorium. The findings were concordant with the previous serial study in 149 adult and children ependymoma samples, which reported that the overexpression of CCND1 predominantly resided at supratentorial location and could predict the relapse in gross-total-resection cases [28]. The incremental expression of CCND1 was demonstrated in the study. However, the expression level of CCND1 did not correlate with 10 years overall survival significantly (p=0.2902) (Supplementary Figure 2). As gross total resection plays an important role in predicting PFS and OS (Table 2), it is possible that CCND1 may play a more important mediator function in the residual ependymomas to facilitate DNA repair and promote residual tumor re-growth after the radiation treatment. Our data also revealed that the radiation treatment upregulated CCND1 mRNA and resulted in strong immunohistochemistry labeling in 3 out of 5 supratentorial and all two infratentorial tumors.

In the radiation-treated ependymomas cell model, CCND1 up-regulated after 6 and 8 Gy irradiation at 7 days. Furthermore, the expression of γH2AX with DNA damage was associated with improved progression-free survival in 15 patients with tissue available [30]. We demonstrated that homologous recombination activities of DNA repair were significantly suppressed after knocking down CCND1and the levels of γH2AX were enhanced after irradiation of ependymoma cells. (Figure 5E & 5F). The phenomenon implied that CCND1-mediated DNA repair may play an important role in treatment failure. On the other hand, previous study showed that CCND1 preferentially associate with CDK4/6 and promote cell proliferation in normal condition. Irradiation triggers the dissociation of CCND1 from CDK4/6, and alternatively interacts with BRCA2 and recruits RAD51 to the DSB site [16]. We suggest that these mechanisms describe why knocking down CCND1 exclusively suppresses DNA repair activity in the irradiated cells rather than the normal condition. The new therapeutic approach by targeting CCND1 translation and expression, such as mTOR inhibitors, or to affect downstream genes expression using CDK4/6 inhibitors, such as palbociclib, will be the upcoming focus for the clinical applications.

Our data declared the limitation of present therapeutic strategy. Gross total resection still stands the most important role on the long-term OS and PFS in the multimodality treatment. The adjuvant irradiation or chemo-radiation can definitely prolong the long-term OS, but not PFS. Incremental expression of CCND1 in primary and recurrent ependymomas, especially on supratentotium, emphasizes the importance in tumor recurrence. Inducing DNA repair by upregulating CCND1 in our ependymoma cell model demonstrates a potential radio-resistance mechanism. Preclinical therapeutic trials combining irradation and cyclin/CDK pathway targeting will be important to test the clinical feasibility.

## MATERIALS AND METHODS

### Clinical data

Following institutional ethical approval, we retrospectively analyzed 82 cases of ependymoma, less than 20 years old, including 2 cases (WHO grade I), 27 cases (grade II) and 53 cases (grade III), who received multimodality treatment in Taipei Veterans General Hospital before June 2012. Clinical data were retrieved from patient charts included age at presentation, gender, tumor location, pathological diagnosis and grading, date of first surgery, extent of resection, adjuvant irradiation and/or chemotherapy use, date of recurrence, supplementary therapy, and date of last follow-up or date of death. For the definition of hazard location, either invasion to four ventricular floor, foramen of Luschka, cerebello-pontine angle or pre-pontine area, lateral medullary cisterns on the pre-operative magnetic resonance images was enrolled. The gross total resection was defined as none residual tumors on the post-operative MR images. The modalities of radiation were mainly conformal intensity modulated radiation therapy (IMRT) 45-59.4Gy post-operatively for patients of older than 2 years of age. During the period of three decades in our series, varied adjuvant chemotherapy protocols had been applied including cisplatin-based regimens, temozolomide and/or intrathecal nimustine (ACNU). The tumor recurrence is defined as an identified new tumors or progression of residual tumors on the imaging study during or after the adjuvant treatment. For focal recurrence, the patients will receive reoperation to eradicate the tumor as much as possible then the gamma knife radiosurgery was applied to enhance local control. However, if the leptomeningeal seeding happened, chemotherapy and/or cranio-spinal re-irradiation were suggested for salvage treatment.

### Immunohistochemistry

According to previous study, the diagnostic criteria for anaplastic ependymomas were the presence of any two of the four parameters, including mitoses > or = 4/10 hpf (1.7/mm2), hypercellularity, endothelial proliferation and necrosis [31]. Staining of Ki-67 of representative area in the 37 cases were reviewed. The MIB-1 (monoclonal, 1: 75, Immunotech, Marseille, France, microwaved three times for 5 min each time) indices were calculated to be positive on tumors with strong staining. Base on the literature, the 5 years overall survival in patients with MIB-1 indices less than 50 and equal or more than 50 was analyzed. Immunohistochemical (IHC) sample preparation and staining for CCND1 and RAD51 were performed as previously described [32]. Antibodyanti-CCND1(cat. No.: GTX112874, 1:2000 dilution, GeneTex, Irvine, CA92606, USA) and RAD51 (cat. No.: GTX118249, 1:2000 dilution,) were used for all IHC experiments.

### Biological samples

The parent/legal guardian of the patients in this study provided informed consent, and all procedures were approved by the Institutional Review Board of VGH-TPE (VGHIRBNU.:2015-12-008A, 20l6-05-007C and 2017-07-001C). Fresh-frozen tumor tissues were collected during surgery in patients with ependymomas. Data of the surgical pathology were retrieved from the Department of Pathology and Laboratory Medicine at Taipei Veterans General Hospital.

### Cell culture and plasmids

Human primary ependymoma cells were obtained from Dr. Seung-Ki Kim & Dr. Ji-Hoon Phi’slab (Seoul National University Children's Hospital, Seoul, Republic of Korea) and Bioresource Collection and Research Center (BCRC, Taiwan). HEK 293T cells and ependymoma cells were maintained in Dulbecco's Modified Eagle Medium (Gibco/Life Technologies, Carlsbad, CA, USA) supplemented with 10% fetal bovine serum (Gibco/Life Technologies). These cells were incubated at 37°C in a humidified atmosphere of 5% CO_2_. For lentiviral expression of shCCND1, plasmid contains shRNA was obtained from the RNAi consortium at Academia Sinica, Taiwan.

### Gene expression microarray (GEM) and computational analyses

Total RNA sample preparation, cDNA probe preparation, array hybridization and data analysis were performed as previously described [33]. Other ependymoma array data were obtained from the Gene Expression Omnibus dataset GSE66354. Micorarray analysis was performed as previously described [33].

### RNA and reverse transcription-quantitative PCR

Total RNAs of tumor tissues and cultured cells were isolated using TRIZOL reagent (Invitrogen/Life Technologies, Carlsbad, CA, USA). Total RNA was reverse-transcribed into complementary DNA through random hexamer priming using a RevertAid First Strand cDNA Synthesis Kit (Thermo Scientific, Waltham, MA, USA). Quantitative PCR was performed in duplicate with gene-specific primers by using a Maxima^TM^ SYBR FAST qPCR kit (Thermo Scientific).

### Homologous recombination detection

Homologous recombination was detected using the Homologous Recombination Assay Kit (Norgen, Thorold, ON, Canada), according to the manufacturer instructions. Briefly, dl-1 and dl-2 plasmids were co-transfected into cells. After 24 hrs, cells were treated with radiation, and then incubated at 37°C for 48 hrs. After the radiation treatment, cells perform homologous recombination (HR) will produce a plasmid recombination amplicon of 420 base pairs (bps) as a result of HR activity. The qPCR fluorescence intensity of amplicon can be directly correlated to the efficiency of HR from the template DNA. DNA were isolated using GeneJET Genomic DNA Purification Kit (Thermo Scientific, Waltham, MA, USA) and were then quantified by qPCR for the homologous recombination activity. The provided assay primer mixtures are designed exclusively for the homologous recombination products but not the original transfected dl plasmids (dl-1 or dl-2). The provided universal primer mixtures were used to detect the original transfected dl plasmids (dl-1 or dl-2) as an internal control. To detect the homologous recombination product, thermal cycler was programmed into 3 minutes at 95°C as initial denaturation, followed by 40 cycles of 15sec at 95°C for denaturation, 30 sec at 61 °C as annealing, 60 sec at 72 °C for extension.

### MTT assay

To evaluate cell viability, cells were seeded at the concentration of 5 × 10^3^/well and incubated at 37°C. After the incubation for 24, 48, 72, and 96 hrs, the cells were treated with 1% thiazolyl blue tetrazolium for 30 min at 37°C followed by 0.1% sodium dodecyl sulfate in 2-propanol, and were mixed thoroughly. The results were obtained by measuring the absorbance at wave lengths of 570 nm and 650 nm using a multiwell scanning spectrophotometer.

### Immunoblotting

Immunoblotting was performed using anti-CCND1 (clone: 92G2, 1:1000 dilution, Cell Signaling, Danvers, MA, USA) and anti-phopho-Histone H2AX (clone: 20E3, 1:1000 dilution, Cell Signaling) antibodies, followed by visualization using horseradish peroxidase-conjugated secondary antibodies and an enhanced chemiluminescence detection system (Merck Millipore, Darmstadt, Germany).

### Statistics analysis

For each clinical factor, 10 years progression-free survival (PFS) and 10 years overall survival (OS) were estimated using the Kaplan Meier method, and the significance tests (α= 5%) were done on the basis of the log-rank test. The clinical variables that had *P* value <0.05on univariant analysis were analyzed by using a Cox proportional hazards model to define relevant prognostic factors in multivariant analysis. For the *in vitro* experiments, two-tailed student's t-tests were used to assess the significance of mean differences. Differences were considered significant at a *p*<0.05. All data are reported as mean ± SD.

## SUPPLEMENTARY MATERIALS FIGURES


