# Supplementary material for: Significance of cyclin D1 overexpression in progression and radio-resistance of pediatric ependymomas
Source: Oncotarget. 2017 Dec 20;9(2):2527–42. doi: 10.18632/oncotarget.23509 (PMC5788657; doi:10.18632/oncotarget.23509)
Supplement: Supplementary file 1 [file oncotarget-09-2527-s001.pdf]

Significance of cyclin D1 overexpression in progression and radio-resistance of pediatric ependymomas

SUPPLEMENTARY METARIALS

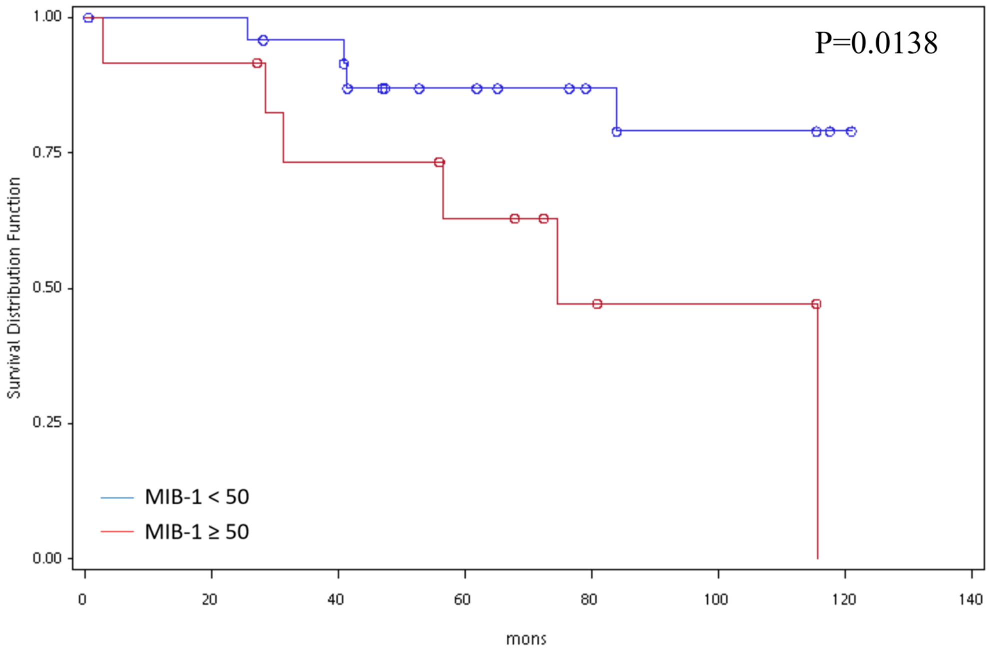

Supplementary Figure 1: The 5 years OS survival for MIB-1 index more or equal to 50 vs. less than 50 were 49% vs 79% (*P*=0.0138).

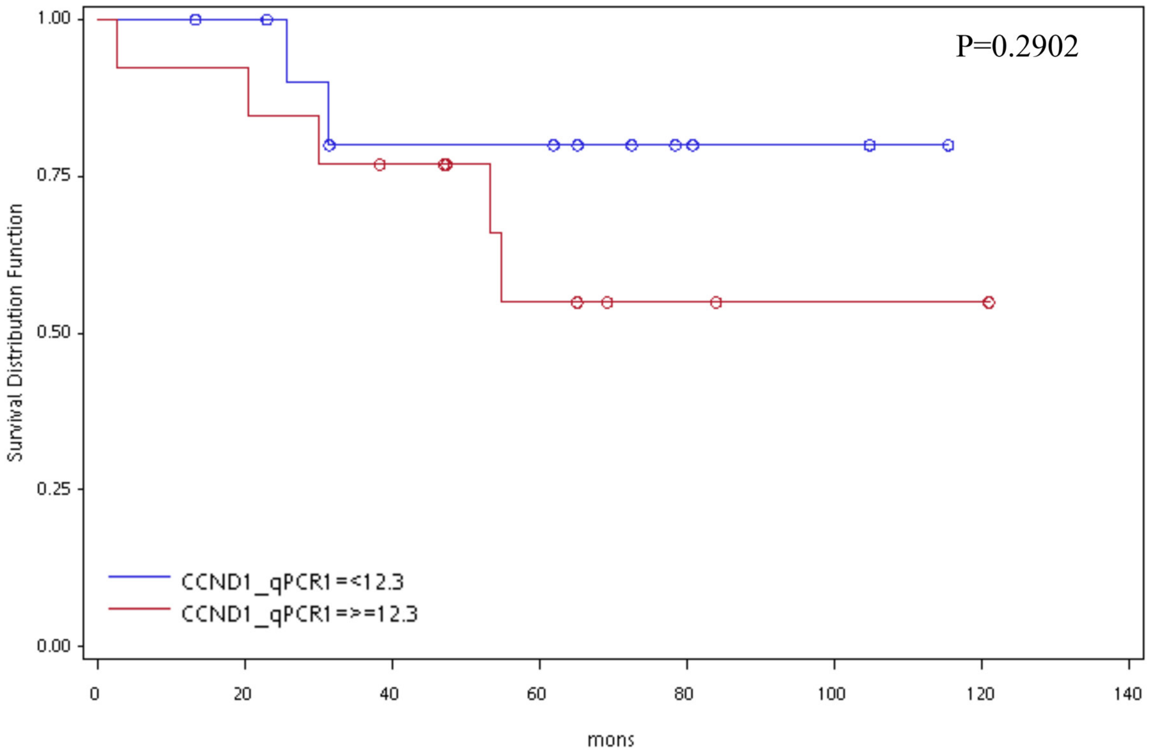

Supplementary Figure 2: The 10 years OS survival for CCND1 expression more or equal to 12.3 (median of qPCR results) vs. less than 12.3. (*P*=0.2902).

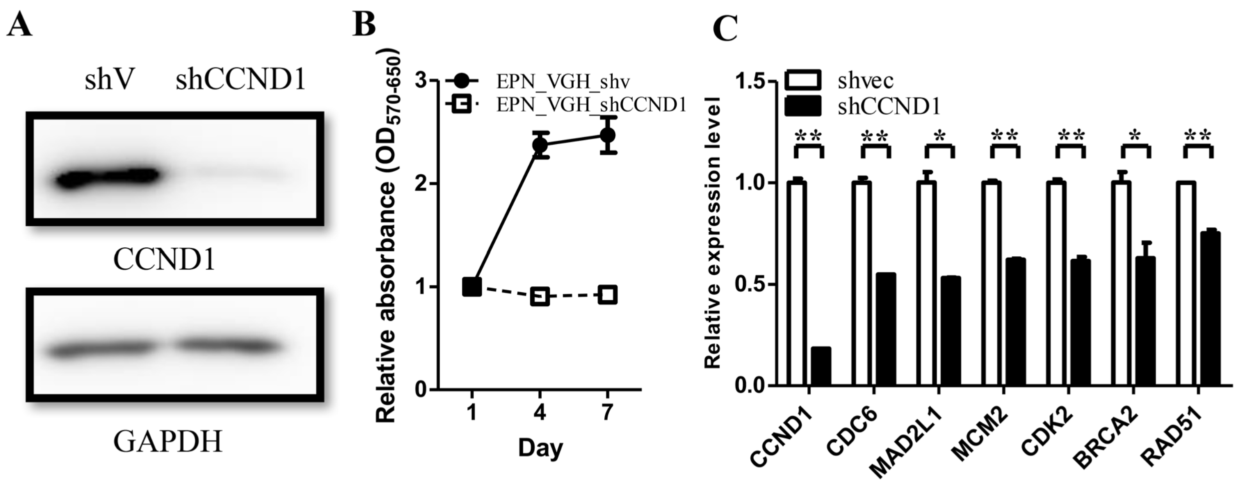

Supplementary Figure 3: (A) Knocking down CCND1 (shCCND1) expression in ependymoma cells, EPN_VGH, which was confirmed through qRT-PCR and immunoblotting. **(B)** Knocking down CCND1 expression decreasedcell proliferation rate in EPN_VGH cells as measured by MTT assay. **(C)** Significant suppression of downstream genes was validated by qRT-PCR. qRT-PCR results are presented as mean±SD for duplicate samples.***p*<0.01 by t-test.
